# Death of Dr. Sims

**Published:** 1883-11-20

**Authors:** 


DEATH OF DR. SIMS.—Dr. J. Marion Sims died in New York, November 13th, 1883. Dr. Sims was a native of South Carolina, but finally settled in New York. He attained to great and deserved eminence in the department of gynecology.

NEW YORK, October 29, 1883.

*To the Editors of the Southern Medical Record.*

DEAR SIRS,—Will you kindly draw the attention of your readers to the following announcement regarding the United States Pharmacopœia :

Any person having purchased a copy of the United States Pharmacopœia of 1880, and desiring a list of the corrections since made therein, can procure the same by sending a two cent stamp to

WILLIAM WOOD & Co., Publishers,
Nos. 56 and 58 LaFayette Place, New York City.

SEE THE INSERT in this issue of THE RECORD of one of the oldest and most reputable manufacturing houses in this country—Wm. S. Merrell Chemical Company, who make a specialty of medicines of choice quality for physicians' use only; and the enormous growth of their business is due to the care used in the selection of all the crude material used, and the accurate methods by which the most perfect preparations are obtained from the large variety of drugs manipulated in their Laboratory. The proprietors claim that their Green Drug Fluid Extracts are not theoretical creations of an over-fertile brain, for money making purposes, but are the results of thirty years of original investigation and scientific research. Messrs. Lamar, Rankin & Lamar, of Atlanta, are general agents for Georgia.

